# TaqMan Real-Time PCR Assays for Single-Nucleotide Polymorphisms Which Identify *Francisella tularensis* and Its Subspecies and Subpopulations

**DOI:** 10.1371/journal.pone.0107964

**Published:** 2014-09-19

**Authors:** Dawn N. Birdsell, Amy J. Vogler, Jordan Buchhagen, Ashley Clare, Emily Kaufman, Amber Naumann, Elizabeth Driebe, David M. Wagner, Paul S. Keim

**Affiliations:** 1 Center for Microbial Genetics and Genomics, Northern Arizona University, Flagstaff, Arizona, United States of America; 2 Translational Genomics Research Institute, Flagstaff, Arizona, United States of America; Albany Medical College, United States of America

## Abstract

*Francisella tularensis*, the etiologic agent of tularemia and a Class A Select Agent, is divided into three subspecies and multiple subpopulations that differ in virulence and geographic distribution. Given these differences, there is a need to rapidly and accurately determine if a strain is *F. tularensis* and, if it is, assign it to subspecies and subpopulation. We designed TaqMan real-time PCR genotyping assays using eleven single nucleotide polymorphisms (SNPs) that were potentially specific to closely related groups within the genus *Francisella*, including numerous subpopulations within *F. tularensis* species. We performed extensive validation studies to test the specificity of these SNPs to particular populations by screening the assays across a set of 565 genetically and geographically diverse *F. tularensis* isolates and an additional 21 genetic near-neighbor (outgroup) isolates. All eleven assays correctly determined the genetic groups of all 565 *F. tularensis* isolates. One assay differentiates *F. tularensis*, *F. novicida*, and *F. hispaniensis* from the more genetically distant *F. philomiragia* and *Francisella-*like endosymbionts. Another assay differentiates *F. tularensis* isolates from near neighbors. The remaining nine assays classify *F. tularensis*-confirmed isolates into *F. tularensis* subspecies and subpopulations. The genotyping accuracy of these nine assays diminished when tested on outgroup isolates (i.e. non *F. tularensis*), therefore a hierarchical approach of assay usage is recommended wherein the *F. tularensis*-specific assay is used before the nine downstream assays. Among *F. tularensis* isolates, all eleven assays were highly sensitive, consistently amplifying very low concentrations of DNA. Altogether, these eleven TaqMan real-time PCR assays represent a highly accurate, rapid, and sensitive means of identifying the species, subspecies, and subpopulation of any *F. tularensis* isolate if used in a step-wise hierarchical scheme. These assays would be very useful in clinical, epidemiological, and/or forensic investigations involving *F. tularensis*.

## Introduction

*Francisella tularensis* is a Gram-negative bacterium that causes the zoonotic disease tularemia in mammals [1] and is a rapidly emerging disease in humans in several regions of the world [2], [3]. It is extremely infectious, with as few as ten organisms capable of causing disease [4]. Respiratory acquired infections are particularly dangerous, having a high mortality in the absence of antibiotic treatment [4]. Due to its virulence and potential for bioweapons development, *F. tularensis* is currently classified as a Class A Select Agent by the Centers for Disease Control and Prevention (CDC) [5]. *F. tularensis* is subdivided into three subspecies: *tularensis*, *holarctica*, and *mediasiatica*. *F. novicida* rarely causes disease and, thus, is rarely isolated [1], [6], but it does share high levels of genomic and biochemical similarity to *F. tularensis*. For this reason, it is often considered a fourth subspecies of *F. tularensis* [7]. This classification remains controversial [8], however, and this paper will treat *F. novicida* as a separate species. The species membership within the *Francisella* genus is rapidly expanding and includes numerous fish pathogens [9], [10], opportunistic human pathogens such as *F. hispaniensis* [7] and *F. philomiragia* [11]–[13], and non-pathogenic *Francisella*-like bacteria found in ticks [14] and soil samples [15].

The three subspecies of *F. tularensis* have distinct virulence, geographic distributions, and host/vector associations. *F. tularensis* subsp. *tularensis* (type A) causes a life-threatening form of tularemia and is found only in North America [1], [16]. *F. tularensis* subsp. *tularensis* is further divided into type A.I and A.II subpopulations, which are correlated with geographically separate host and vector distributions [17]. These subpopulations also appear to differ in virulence, with A.I associated with more severe disease than A.II [16], [18]. Subpopulation A.I is further differentiated into numerous smaller subpopulations associated with differing disease severity [19], [20] and geographical distribution [21]. *F. tularensis* subsp. *holarctica* (type B) is further separated into Japanese (sometimes referred to as biovar *japonica*) and non-Japanese groups due to biochemical and genetic differences distinguishing Japanese type B isolates from the other type B isolates found throughout the northern hemisphere [1]. *F. tularensis* subsp. *mediasiatica* is geographically restricted, having been isolated only from central Asia [1].

Numerous molecular typing methods are available but each has specific limitations due to either high cost (labor and expertise) and or a narrow range of discrimination within *F. tularensis* subspecies and principle populations. Molecular typing methods such as pulsed-field gel electrophoresis (PFGE), amplified fragment length polymorphism (AFLP) [22], RD1 [23], and multi-locus variable-number tandem repeat (VNTR) analysis (MLVA) [24] have been shown to be highly effective at *F. tularensis* species and subspecies differentiation [1]. However, these molecular methods are expensive, labor intensive, and often require extensive experience to perform [1], making them ill-suited for clinical diagnostics. Simple presence/absence PCR-based assays are effective at confirming *F. tularensis* from clinical [25]–[28] or environmental samples [14], [29]–[32] and can be highly sensitive when a TaqMan fluorogenic real-time PCR platform is used [29], [32]–[36], but they cannot further differentiate among the subspecies or subpopulations nor can they differentiate a negative result from a PCR failure. Other PCR assays do provide genetic differentiation at varying resolution [37], [38], some between *F. tularensis* subspecies *tularensis* and *holarctica* [33], [34], [39], [40] and another includes *mediasiatica* [41]. Other assays are capable of differentiating subpopulations of *F. tularensis* subsp. *tularensis* subpopulations A.I and A.II [16], [42]. Several studies have presented SYBR green based real-time PCR assays that differentiate numerous genetic groups within *F. tularensis* [19], [43], [44]. However, due to the mild inhibitory properties of SYBR green [45], these assays are not typically highly sensitive to samples containing low level DNA amounts or trace PCR inhibitors, which are typical of environmental and clinical samples [46]. Although SYBR assays are ill-suited for environmental and clinical samples, Dual Probe TaqMan real-time PCR assays have been successfully used on such samples due to their sensitivity to low level DNA amounts [16], [47] and tolerance for the trace amounts of PCR inhibitors. However, there is no simple assay or set of assays available, in the form of highly sensitive TaqMan real-time PCR assays, that are capable of distinguishing the species *F. tularensis* and then further differentiating *F. tularensis* isolates into all of the known subspecies and principle subpopulations. Such differentiation is highly desirable given the differences in virulence and geographic distributions among these major genetic groups [1], [48]. Obtaining this information would be a necessary first step in any epidemiological or forensic investigation involving *F. tularensis*, which would likely involve environmental or clinical samples.

Single nucleotide polymorphisms (SNPs) can be highly effective as molecular markers for identifying genetic groups. In clonally reproducing bacterial populations with a low level of horizontal gene transfer, SNPs are highly stable and exhibit little to no homoplasy. Because of this, single SNPs known as canonical SNPs (canSNPs) can be effectively used to define different genetic groups, whether species, major clades (e.g., subspecies), or even individual strains [49]. These canSNPs are also amenable to a variety of high-throughput genotyping methods, including real-time PCR. This strategy has been successfully used for *Brucella* spp. [50], *Yesinia pestis* [51], and *Bacillus anthracis* [52] and is also appropriate for *F. tularensis*, given its clonal nature [53].

**Figure 1:**
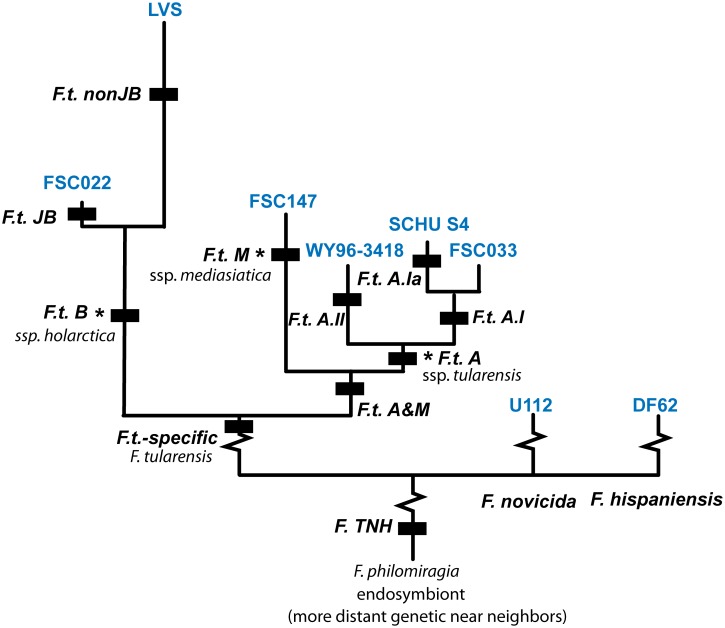
Schematic evolutionary tree of *Francisella tularensis* and *Francisella* genetic near neighbor species. Black bars indicate the important canSNP signatures specific to major genetic groups among *Francisella* species and within *F. tularensis*. The three recognized subspecies*, as well as divisions within the two major subspecies, *tularensis* and *holarctica*, are indicated. The strain representing each genetic group is indicated in blue text.

We present eleven rapid and highly-sensitive TaqMan real-time PCR canSNP genotyping assays that are diagnostic for major branches in the *Francisella* species and *F. tularensis* phylogenies, including: the separation of *F. tularensis*, *F. novicida* and *F. hispaniensis* from the more genetically distant *F. philomiragia* and *Francisella*-like tick endosymbionts; the branches leading to the three official *F. tularensis* subspecies; and numerous principal populations (Figure 1). We designed TaqMan real-time PCR assays for the canSNPs defining these lineages and tested the specificity of the canSNP-signatures by running the assays across a large genetically and geographically diverse DNA panel.

## Methods

We identified SNPs that were potentially specific to subpopulations within *F. tularensis* and the closely related species *F. philomiragia* and the *Francisella*-like endosymbionts based on a survey of numerous publications [15], [19], [43], [44], [54] and an in-house sequencing effort on ParC gene from a single endosymbiont-infected tick submitted to GenBank (GQ303199). Eleven of these SNPs were used to develop TaqMan real-time PCR canSNP assays targeting the following *Francisella* genetic groups: (Figure 1) *F. tularensis*, *F. novicida* and *F. hispaniensis* (*F*. TNH), *F. tularensis-*specific (*F.t.-*specific), *F. tularensis* subspecies *tularensis* and *mediasiatica* (*F.t.* A & M), *F. tularensis* subsp. *mediasiatica* (*F.t.* M), *F. tularensis* subsp. *tularensis* (*F.t.* A), *F. tularensis* subsp. *tularensis* subpopulation A.I (*F.t*. A.I), *F. tularensis* subsp. *tularensis* subpopulation A.Ia (*F.t*. A.Ia), *F. tularensis* subsp. *tularensis* subpopulation A.II (*F.t*. A.II), *F. tularensis* subsp. *holarctica* (*F.t.* B), Japanese *F. tularensis* subsp. *holarctica* (*F.t.* JB) and non-Japanese *F. tularensis* subsp. *holarctica* (*F.t.* nonJB).

Primers (Integrated DNA Technologies, San Diego, CA) and allele-specific TaqMan -minor groove binding (MGB) probes (Applied Biosystems, Foster City, CA) targeting each canSNP were designed using Primer Express software (Applied Biosystems) (Table 1). Each 5 µl TaqMan real-time PCR canSNP assay reaction contained 1× TaqMan Universal PCR master mix (Applied Biosystems), primers and probes (for concentrations see Table 1), and 1 µl diluted DNA template. Additionally, the *F.t.* A and *F.t.* A.II canSNP assays were supplemented with 0.025 U/µl of Platinum *Taq* DNA polymerase (Invitrogen) for improved efficiency. The TaqMan real-time PCR canSNP assays were run on an Applied Biosystems 7900HT Fast Real-Time PCR System with SDS software version 2.3 under the following conditions for all assays except *F.t.* JB: 50°C for 2 min, 95°C for 10 min, and 50 cycles of 95°C for 15 sec and 60°C for 1 min. The *F.t.* JB canSNP assay followed identical conditions except for an annealing temperature of 61.5°C.

**Table 1:** Primers and TaqMan-MGB probes for *F. tularensis* canSNP assays.

Specificity	Branch	SCHU S4 Genome position	Primer Sequences (5′à3′)^a^	Primer (µM)^b^	Probe Sequences (5′à3′)^c^	Probe(µM)^d^	SNPidentity^e^	AnnealingT_m_°C^f^	SNPsource
									
*F.*TNH	F.1	397,255	F TTACWMRATTACCTCATCARGTTTCAAGTG	0.9	D VIC-AATGGAGCAAATcGCTAA	0.20	**C**	60	this study
			R ATATTTTTWATCCAWGTRATYTTYTGTTGC	0.9	A 6FAM-AATGGAGCAAATtGCTAA	0.20	T		
*F.t*.-specific	T.1	1165688	F CTAAGCCATAAGC CCTTTCTCTAACTTGT	0.9	D 6FAM-CTTTTGAACgCTTGACAT	0.20	G	60	Svensson et al. 2009
			R AGCAATGACAAA GCTTGTTGAAAAAG	0.9	A VIC-CCTTTTGAACaCTTGACAT	0.20	A		
*F.t.* M	M.1	75,124	F GGACCGGGCATGCTCTT	0.9	D VIC-CAGGGTAAtTTAGCG	0.20	T	60	this study
			R GCAAACCACTCC GTATAGAAATCA	0.9	A 6FAM-CAGGGTAAcTTAGCGC	0.20	**C**		
*F.t.* A&M	A/M.1	1,491,914	F TGATTGTCTCTAGC TCCAACATAGACA	0.9	*D 6FAM-AAGCTAGAtAAAGCT	0.20	**A**	60	this study
			R GGCACATACACTC TTAGGAAAGCA	0.9	*A VIC-AAGCTAGAcAAAGCT	0.20	G		
** *F.t.* A	A.1	397,639	F TTCAGCCTGGATT TCAGAAAGTGT	0.9	*D VIC-CCACTTGaATCATCA	0.20	**T**	60	this study
			R CAGACTAGTTTGGA TAAGGTTTTAGATCGT	0.9	*A 6FAM-CCACTTGgATCATCA	0.20	C		
*F.t.* A.I	A.I.1	75,109	F TGCGCAGCAGCTGATAGG	0.9	D VIC-TTTACATACaCTGTATCAGG	0.20	**A**	60	this study
			R ACCACTCCGTATAG AAATCAGTTTTGT	0.9	A 6FAM-TTACATACgCTGTATCAG	0.20	G		
*F.t.* A1a	A.I.12	142781	F TGGCAAAAAATACTT ATGGTACGGGTT	0.9	*D VIC-CATTCATCAA**g**GCAAAAC	0.25	C	60	Pandya et al. 2009
			R ACCTTCATCTGAAT AAACTGGCTTATCG	0.9	*A 6FAM-ATTCATCAAcGCAAAAC	0.25	G		
** *F.t.* A.II	A.II.1	84,150	F GTTTAATTGGTGGCGCATCTTTGA	0.9	*D VIC-CCGTACATATCTTaTTTGCT	0.20	T	60	this study
			R CGCAATAGCTGCGAT AATATCAATAGTTAAAA	0.9	*A 6FAM-CGTACATATCTTgTTTGCT	0.20	**C**		
*F.t.* B	B.1	83,745	F AGAGAAGATCTCTAT TTGCTGAGTCTGA	0.9	D VIC-AGCTTAACAAaATTATAG	0.20	A	60	this study
			R CTATCATCTAGTGATT CACCAATACACACT	0.9	A 6FAM-AAGCTTAACAAgATTATA	0.20	**G**		
*F.t.* JB	B.16	608,245	F CTATATAACCACCATC CAAAGATTTAGCT	0.9	*D 6FAM-CATAGCATAAGaCTTTTGAT	0.10	T	61.5	Svensson et al. 2009
			R ACCGCTTGAAAAGTTAGGTATGCT	0.9	*A VIC-CATAGCATAAGcCTTT	0.25	**G**		
*F.t.* nonJB	B.2	5,162	F AATTGAAGCACGCCAAAAATAG	0.9	*D VIC-CTGTAGTAAtTACAAGGCT	0.20	A	60	Vogler et al. 2009
			R GCAACTTTWGGGATGATTTTAGC	0.9	*A 6FAM-CTGTAGTAAgTACAAGGCT	0.20	**C**		

a F: forward primer, R: reverse primer.

b Primer concentration (µM) per reaction.

c D: derived probe, A: ancestral probe, lower case bolded text within the probe sequence indicates SNP.

*Probes designed on the reverse complement.

d Probe concentration (µM) per reaction.

e SNP states relative to the SCHU S4 whole genome sequence position (NC_006570).

f Real-time PCR annealing temperature.

**Supplemented with 0.025 U/µl of Platinum *Taq* DNA polymerase (Invitrogen) for improved efficiency.

We confirmed the specificity of all eleven SNP signatures by screening the canSNP assays across a panel of 586 genetically and geographically diverse *Francisella* DNAs, including: 82 *F. tularensis* subsp. *tularensis* subpopulation A.I, 33 *F. tularensis* subsp. *tularensis* subpopulation A.II, 446 *F. tularensis* subsp. *holarctica* (including 7 from Japan), 4 *F. tularensis* subsp. *mediasiatica*, and 21 genetic near-neighbor strains (8 *F. novicida*, 1 *F. hispaniensis*, 2 tick endosymbionts, and 10 *F. philomiragia*) (Table S1). The *F.t.*-specific and *F.*TNH-specific assays were also screened across an additional 7 environmental tick samples positive for endosymbionts (data not shown). The *F.*TNH-specific assay separates *F. tularensis*, *F. novicida* and *F. hispaniensis* from the more genetically distant *F. philomiragia* and *Francisella*-like tick endosymbionts). Two negative controls per canSNP assay were included with each experiment. The DNAs consisted of whole genome amplification (WGA) products (Qiagen, Valencia, CA) and genomic DNA from various types of DNA preparations (heat soaks, chloroform, and Qiagen DNA extractions). All of the WGA products were prepared from DNAs extracted from pure culture except for the two tick endosymbiont WGA products, which were amplified from starting DNA material that had been extracted from an *Francisella*-like endosymbiont infected tick (i.e., contained both tick endosymbiont and tick DNA). DNA templates were diluted prior to amplification in the canSNP assays at these ratios: 1∶49 for WGA products, 1/10 for heat soak, and 1/100 for Qiagen or cholorform extractions. We tested the sensitivity of each canSNP assay by running it across four replicates of a serial ten-fold dilution series (10^−1^–10^−10^) of WGA products or genomic DNA from two DNA templates, one that possessed the targeted genetic group -specific (i.e. derived) allele and one that possessed the alternate (i.e. ancestral) allele.

## Results and Discussion

All eleven canSNP assays displayed robust signal amplification from the perfect match probe with the mismatched probe signal either failing or showing weaker fluorescence. Six canSNP assays, *F.* TNH, *F.t.* M, *F.t.* A.I, *F.t.* A.II, *F.t.* JB, and *F.t.* nonJB showed some cross-hybridization with the mismatched probe but the delta C_T_ values of the match and mismatched probes were sufficient (≥4.7 C_T_ when analyzed at a threshold of 0.2) to provide clear genotyping (data not shown). Assay performance was equally robust regardless of the template DNA preparation method.

Our *F*. TNH canSNP assay detects *F. tularensis* and several members of the *Francisella* species that are common genetic near neighbors. The *F.* TNH canSNP assay differentiated *F. tularensis* and their closer genetic-near neighbors, *F. novicida* and *F. hispaniensis*, from the more distant genetic near-neighbors the *Francisella*-like tick endosymbionts and *F. philomiragia* (Fig. 1). Specifically, the 565 *F. tularensis*, 8 *F. novicida*, and 1 *F. hispaniensis* DNAs displayed the derived (*F*. TNH-specific) allele and the genetic near-neighbors (9 tick endosymbionts and 9 of 10 *F. philomiragia*) DNAs displayed the ancestral (alternate) allele (Table S2). A single *F. philomiragia* DNA failed to amplify, likely due to additional SNPs within the primer/probe sites, although we were unable to confirm this due to a lack of available sequence. We did, however, confirm the ancestral SNP state for this DNA by using a different genotyping assay targeting the same SNP (data not shown). Differentiation among near neighbors appears to be based on the phylogenetic distance among strains but this hypothesis cannot be confirmed due to a lack of whole-genome sequence data for *F. hispaniensis* and the tick endosymbionts.

The *F.t.*-specific canSNP assay correctly differentiated *F. tularensis* species from their genetic near-neighbors *F. novicida* and *Francisella*-like tick endosymbionts (Figure 1). Specifically, the 565 *F. tularensis* DNAs displayed the derived (*F.t.-*specific) allele and the genetic near-neighbors (8 *F. novicida* and 5 tick endosymbionts) DNAs displayed the ancestral (alternate) allele (Table S2). The assay failed to amplify on some strains of *Francisella*-like tick endosymbionts (4 out of 9) and the other near-neighbor species, *F. hispaniensis* and *F. philomiragia* DNA samples. Amplification failure on these DNAs is likely due to additional SNPs within the primer/probe sites, although we were unable to confirm this due to a lack of available sequence. Our *F.t.*-specific canSNP assay is highly specific to *F. tularensis*, indicated by the detection and correct (derived) genotype of all 565 *F. tularensis* samples, but fails to amplify some genetic near neighbors within *Francisella* species. Despite this limitation, the genotype calls among those detected near-neighbor samples were consistently accurate (ancestral).

To have a robust *F.t.-specific* assay that definitively identifies *F. tularensis* DNA and differentiates it from or completely fails on samples that are close genetic near neighbors is highly desirable because *F. tularensis* (the primary source of tularemia) is a class A select agent [5]. Numerous *Francisella* species with cryptic ecologies abundantly exist in the environment [7], [11], [14], [15], [55] and have triggered false positive signals in molecular detection systems intended for the surveillance of *F. tularensis* [15]. These detection systems were designed on molecular signatures thought to be specific to *F. tularensis*. Our extensive validation studies strongly suggest that our *F.t.*-specific assay may be able to correctly differentiate unculturable *Francisella* genetic near-neighbors in the environment that have caused problems with the national BioWatch monitoring program [15], [56] from *F. tularensis.* However, we were, unfortunately, unable to obtain any of these environmental samples for testing our assay against this type of background.

The ability to detect genetic-near neighbors in addition to differentiating them from *F. tularensis* serves to improve our understanding of *F. tularensis* by providing a better understanding of its nearest genetic relatives. The ability of *F.* TNH assay to detect the non-select agent forms of *Francisella* species, disease causing or benign, can be highly useful in this investigative endeavor. For example, the combined usage of our *F*.*t.*-specific and *F.* TNH canSNP assays permit the detection of *F. tularensis* and tick endosymbionts while correctly differentiating *F. tularensis* from tick endosymbionts. This is particularly useful in nature because *F. tularensis* is commonly associated with ticks [14], thus making ticks a likely background in which to find *F. tularensis* DNA. However, ticks are also likely to be carrying tick endosymbionts, which are genetic near-neighbors to *F. tularensis* [14], [57]. Tick samples positive for both endosymbionts and *F. tularensis* presented as a mixture with *F.t*.-specific and *F*. TNH canSNP assays, with amplification of both the specific and alternate alleles (data not shown) with significantly diminished net C_T_ difference when compared to homogenous DNA samples (data not shown). This net C_T_ difference between mixed vs homogenous DNA samples allowed for a statistically significant means of identifying *F. tularensis* even in a background containing closely related tick endosymbionts. This ability makes the *F.t.*-specific and *F*. TNH canSNP assays, when used in combination, an accurate and simpler means of differentiating *F. tularensis* from tick endosymbionts.

*F. tularensis* isolates are comprised of numerous subpopulations [16], [43], [44] that have differences in geographic distribution and associated virulence [1], [18]–[20], [48]. For these reasons, *F. tularensis*-specific assays that can further differentiate a given *F. tularensis* sample into its subspecies and subpopulation classifications are especially useful for the study of this organism. Our nine *F.t.* subspecies and subpopulation canSNP assays correctly assigned *F. tularensis* DNA from our *F. tularensis* diversity panel (n = 565; Table S1) to their known genetic group (subspecies or subpopulation) (Fig. 1 & Table S2). Each canSNP assay genotyped a *F. tularensis* DNA into a particular genetic group (represented as possessing the derived allele state) or the alternate group (possessing the ancestral allele state) (Table S2), thus making these nine canSNP assays ideally suited to further subgroup *F. tularensis* DNA samples.

In contrast to their excellent performance on *F. tularensis* DNA samples, our nine *F. tularensis* subpopulation canSNP assays are not ideally suited to be used on genetic near neighbors of *F. tularensis* (*F. hispaniensis*, tick endosymbionts and *F. philomiragia*). These nine assays were designed accounting for the sequence variation found among *F. tularensis* genomes and not near neighbors. The genetic distance between *F. tularensis* and near neighbors [15] increases the chances of additional SNPs in the primer or probe sequence regions targeted by the assays. Therefore, it was not surprising to find confounding results such as amplification failures, loss of probe specificity, and/or homoplasy when these assays were screened on near-neighbor samples. Particular issues appeared to be specific to assays and the tested near-neighbor strain DNA sample (Table S2). All the assays, except *F.t.* A.II, failed to amplify the endosymbiont-infected tick samples (Table S2). *F.t.* A & M, *F.t.* B, and *F.t.* JB failed to amplify the *F. hispaniensis* sample. *F.t.* A & M consistently failed to amply *F. philomiragia* samples although all other assays displayed sporadic amplification for this distant genetic group. Among the sporadically amplifying assays, PCR amplification of *F. philomiragia* was significantly compromised as indicated by delays in PCR amplification (data not shown). *F.t.* AI, *F.t.* B, and *F.t.* nonJB display a loss of probe specificity, which appears as the reporting of both alleles or a conflict of allele calls among replicates of amplified *F. philomiragia* samples. Four assays resulted in homoplastic assignment of near neighbor strains by genotyping them as the derived allele state (genetic group-specific). *F.t.* A.II, *F.t.* B, and *F.t.* nonJB homoplastically genotyped the *F. philomiragia* samples and *F.t.* M homoplastically genotyped the *F. hispaniensis* sample (Table S2). In addition, when we *in silico* genotyped the *F. philomiragia* whole genome sequenced strain 25017 (GeneBank accession NC_010336), we also observed homoplastic genotyping results for the *F.t.* A&M, *F.t.* M, *F.t.* A.II and *F.t.* B canSNP assays, although *F.t.* M amplified as ancestral allele state *in vitro* albeit in a sporadic manner. This conflict between *in silico* data and *in vitro* result could be due to sequencing errors under the probe site or homoplastic genotyping by *F.t.* M due to neighboring base mismatches near the SNP site. The sporadic amplification of *F.t.* M assay on *F. philomiragia* samples suggest the later. Based on our extensive validation study, our nine subpopulation canSNP assays will provide accurate data on only *F. tularensis* samples. Therefore, we recommend to use the nine *F.t.* subpopulation canSNP assays only once the DNA sample is confirmed as *F. tularensis*.

The above results suggest that the eleven canSNP assays presented here could be used in a step-wise fashion to identify any unknown sample potentially containing *Francisella*. Specifically, any such unknown sample could first be screened using our *F.t.*-specific canSNP assay to definitively distinguish *F. tularensis* from its genetic near-neighbors. A sample that possesses the derived allele (*F.t.*-specific) could subsequently be screened across our nine *F. tularensis* subspecies and subpopulation canSNP assays. Our validation data suggest that this would result in the accurate classification of the confirmed *F. tularensis* sample into subspecies and subpopulation. If all eleven assays are used concurrently on an unknown sample for efficiency purpose, then the validity of nine *F. tularensis* subpopulation assays will be dependent on the *F. tularensis* status of the examined sample. It is critical that only samples that are derived for *F.t.*-specific assay be examined on the nine *F. tularensis* subspecies and subpopulation canSNP assays because these assays were designed accounting for *F. tularensis* genomes and not near neighbors. Also, our validation study showed sporadic amplification, loss of probe specificity and homoplastic results when these assays were screened on genetic near-neighbors. Samples that result as possessing the ancestral allele (alternate) or fail altogether on *F.t.-*specific canSNP assay can be screened using our *F.*TNH canSNP assay to determine the presence of other *Francisella* species and possibly differentiate among the *F.* TNH-specific and alternate near-neighbor *Francisella* species. The sporadic amplification, loss of probe specificity, and homoplastic results described among the nine *F. tularensis* subspecies and subpopulation canSNP assays also illustrate the importance of validating potential canSNPs across a large panel of isolates that includes genetic near-neighbors, particularly genetic near-neighbors that might be found in close proximity to the target.

Our eleven canSNP assays also showed considerable sensitivity in detecting and genotyping low concentrations of WGA DNA. Results from ten-fold serial dilution experiments indicated that these canSNP assays are able to consistently detect and genotype *F. tularensis* WGA DNA at dilutions as low as 10^−3^ to 10^−7^, depending on the assay. Sporadic amplification was observed at dilutions 10^−4^ to 10^−9^, depending upon the canSNP assay (data not shown). All eleven assays completely failed on negative water controls, furthering our confidence in the specificity and sensitivity of our assays to detect low concentrations of DNA targets. WGA products possess unequal copies of different loci across the genome due to amplification bias inherent to the WGA process [58]. Therefore, the total DNA concentration or copy number of a locus in a WGA sample cannot be accurately calculated. However, in our serial dilution experiments, the C_T_ values ranged from 29–48 in the dilutions where low level but consistent amplification was observed, depending upon the canSNP assay. These C_T_ values are comparable to the C_T_ values for other assays run against templates containing 100 fg of DNA [50], [51]. This comparison suggests that our *F. tularensis* canSNP assays have the same level of sensitivity as other TaqMan MGB real-time PCR dual probe assays [50], [51] and likely have consistent detection of ∼100 fg and sporadic detection of ∼10 fg of target genomic DNA. The ability to detect and genotype low levels of target DNA makes these canSNP assays highly valuable diagnostic tools for use in clinical, epidemiological, and/or forensic investigations where samples often suffer from low DNA concentrations and/or limited quantities.

The eleven TaqMan real-time PCR canSNP assays presented here provide a simple means of obtaining highly accurate and sensitive typing scheme that classifies unknown isolates to species, subspecies, and subpopulation level when used or examined in a progressive, step-wise fashion. The real-time platform is amenable for high throughput screening at a rapid pace, permitting classification within 3 hours. Our eleven assays, as a collection, provide a comprehensive genotyping scheme that overcomes specific limitations that burden other published individual typing schemes. As mentioned previously, the virulence and geographic distribution differences among *F. tularensis* genetic groups [1], [19], [20] would make such identification a logical first step in any investigation. In clinical investigations, species, subspecies, and subpopulation identification would be useful for diagnosis and predicting disease outcome as well as for assessing risk associated with handling clinical isolates [59]. These genetic tools will also assist epidemiological investigations, particularly within the United States, where *F. tularensis* subsp. *tularensis* subpopulations A.I, A.I.12 and A.II and *F. tularensis* subsp. *holarctica* all occur [1], [17]–[19], [21]. The precise ecology of these different *F. tularensis* genetic groups is still relatively unknown [1] and epidemiological investigations focusing on transmission patterns would benefit from a simple means of differentiating subspecies and subpopulations when analyzing potential source isolates and comparing them to clinical isolates. The Class A Select Agent status of *F. tularensis* also means that any suspected tularemia case may be subject to bioterrorism assessment and a forensic investigation in which molecular subtyping will likely play a significant role [60]. Identifying the subspecies and subpopulation of any forensic isolate will be a necessary first step in any such forensic investigation. Given that we confirmed that these canSNP assays accurately genotyped complex clinical samples (data not shown), consistent with similar successes in other studies [50], [61], we do not foresee problems in adapting these canSNP assays to a clinical or forensic setting. In support of this, *F.t*.-specific and *F.* TNH canSNP assays showed success when directly screened on tick environmental samples. Here, endosymbiont DNA was detected despite high concentration of background Tick DNA (data not shown). In summary, these canSNP assays should have broad applicability for clinical, epidemiological, and forensic applications involving *F. tularensis*.

## Supporting Information

Table S1 **Strain Table.**
(XLS)

Table S2 **canSNP Table.**
(XLSX)
